# Evidence basis for using dexmedetomidine to enhance the quality of paravertebral block: A systematic review and meta-analysis of randomized controlled trials

**DOI:** 10.3389/fphar.2022.952441

**Published:** 2022-09-29

**Authors:** Rong Tang, Yu-Qian Liu, Hai-Lian Zhong, Fang Wu, Shi-Xiong Gao, Wei Liu, Wen-Sheng Lu, Ying-Bin Wang

**Affiliations:** Department of Anesthesiology, Lanzhou University Second Hospital, Lanzhou, China

**Keywords:** dexmedetomidine, adjuvant, paravertebral block, meta-analysis, local anaesthesia (LA)

## Abstract

**Background:** Dexmedetomidine is considered an adjunct to local anaesthesia (LA) to prolong peripheral nerve block time. However, the results from a previous meta-analysis were not sufficient to support its use in paravertebral block (PVB). Therefore, we performed an updated meta-analysis to evaluate the efficacy of dexmedetomidine combined with LA in PVB.

**Methods:** We performed an electronic database search from the date of establishment to April 2022. Randomized controlled trials (RCTs) investigating the combination of dexmedetomidine and LA compared with LA alone for PVB in adult patients were included. Postoperative pain scores, analgesic consumption, and adverse reactions were analyzed.

**Results:** We identified 12 trials (701 patients) and found that the application of dexmedetomidine as a PVB adjunct reduced the postoperative pain severity of patients 12 and 24 h after surgery compared to a control group. Expressed as mean difference (MD) (95% CI), the results were −1.03 (−1.18, −0.88) (*p* < 0.00001, I^2^ = 79%) for 12 h and −1.08 (−1.24, −0.92) (*p* < 0.00001, I^2^ = 72%) for 24 h. Dexmedetomidine prolonged the duration of analgesia by at least 173.27 min (115.61, 230.93) (*p* < 0.00001, I^2^ = 81%) and reduced postoperative oral morphine consumption by 18.01 mg (−22.10, 13.92) (*p* < 0.00001, I^2^ = 19%). We also found no statistically significant differences in hemodynamic complications between the two groups. According to the GRADE system, we found that the level of evidence for postoperative pain scores at 12 and 24 h was rated as moderate.

**Conclusion:** Our study shows that dexmedetomidine as an adjunct to LA improves the postoperative pain severity of patients after surgery and prolongs the duration of analgesia in PVB without increasing the incidence of adverse effects.

## Introduction

The prevalence of paravertebral block (PVB), which has an extensive evidence base as part of a multimodal analgesic strategy for thoracic, breast, and abdominal surgery, may be attributed to technical improvements and its enhanced efficacy and safety. With the continuous development of visualization technology and the wide application of ultrasound technology in clinical anaesthesia, multimodal analgesia dominated by nerve block technology has become a new trend in perioperative analgesia (Schnabel et al., 2010; Tahiri et al., 2011; El-Boghdadly et al., 2016). However, the postoperative analgesic advantage is limited by the duration of local anesthetic (LA). The duration of postoperative analgesia after a single-shot injection may be maintained for 8–12 h even when medium-acting LA and long-acting LA are used (Ding et al., 2018). Although continuous infusion may prolong the analgesia effect, the small paravertebral space makes catheterization difficult and easily displaced. Moreover, it will increase the incidence of pneumothorax, hypotension, nausea, and vomiting (D'Ercole et al., 2018). Therefore, it is recommended to use single injection in clinical application to reduce the occurrence of corresponding complications. As a result, more and more researchers are working on LA adjuncts to improve the duration of postoperative analgesia, and finding suitable and safe LA adjuvants to extend the benefits of analgesia is a relatively simple method proposed in recent years (Opperer et al., 2015).

Dexmedetomidine is a highly effective and selective a_2_-adrenergic receptor agonist with good sedative, hypnotic, and sympathetic blocking effects (Kamibayashi and Maze, 2000). Dexmedetomidine potentiates the inhibition of neuronal conduction and produces analgesia by blocking hyperpolarization-activated cation currents (Brummett et al., 2011). A recent meta-analysis provided strong evidence that perineural dexmedetomidine improved brachial plexus block onset, quality, and analgesia, and moderate evidence supports its role in accelerating the onset of blockade and prolonging the duration of analgesia (Vorobeichik et al., 2017). Although an earlier systematic review of dexmedetomidine as an adjunct to PVB demonstrated its efficacy, there was significant clinical and statistical heterogeneity, and the further subgroup and sensitivity analyses failed to identify the source of heterogeneity, which may undermine the accurate estimation of the dexmedetomidine treatment effect (Wang et al., 2018). Other trials of dexmedetomidine as a PVB adjunct have been published. Therefore, we performed a systematic review and meta-analysis of published studies to evaluate the role of dexmedetomidine combined with LA in PVB.

## Methods

We registered the current meta-analysis at PROSPERO (CRD42022327756). The procedures and methods for this article were based on the criteria of the PRISMA statement guidelines (Moher et al., 2009) and the recommendations of the Cochrane Collaboration (Bero and Rennie, 1995). Randomized trials examining the effect of dexmedetomidine as an adjunct on analgesia after PVB were evaluated using a predefined protocol.

### Literature search

Two of the authors (TR and LYQ) independently searched relevant studies from electronic databases including PubMed, Embase, Web of Science, Clinical Trials, Cumulative Index of Nursing and Allied Health Literature (CINAHL), Latin American and Caribbean Health Sciences Literature (LILACS), Cochrane Library, and Cochrane Central Register of Controlled Trials. The last retrieval date was 1 July 2022. Medical subject headings (MeSH), text words, and controlled vocabulary terms associated with dexmedetomidine, medetomidine, and DEX were searched. These results were combined with the search terms paravertebral block using the Boolean operator “AND”. Our study was limited to RCTs published between the inception of the databases and July 2022. Only trials including adults (age > 18 y) were considered.

### Eligibility criteria

We included randomized trials that evaluated the effects of dexmedetomidine as an adjunct to LA on pain scores and side effects after PVB compared with LA alone. The following specific standards were used:

#### Inclusion criteria

(1) The study type was RCT.
(2) The subjects were adults (age > 18 y) with no gender restriction.
(3) The study design was RCT of dexmedetomidine combined with LA compared with LA alone at any level of PVB for ipsilateral surgeries.

#### Exclusion criteria

(1) The subjects were healthy volunteers.
(2) Paravertebral catheters.
(3) The study design was a comparison between LA with dexmedetomidine and LA with other drugs.
(4) Non-perineural routes of dexmedetomidine administration were used.
(5) The study was ongoing, and the complete data were not available.

### Trial selection and methodological assessment

Two members of the research group (TR and LYQ) independently evaluated the titles, abstracts, and full texts to exclude studies that were irrelevant to the inclusion criteria. When there were disagreements on the inclusion or exclusion of the study, the inconsistencies were resolved by re-evaluating the full article of the source studies and consulting with the independent third researcher (ZHL) until consensus was reached.

Two researchers (TR and LYQ) independently evaluated the methodological quality of the included RCTs based on the risk of bias tool (RoB2) (Higgins et al., 2011). The tool evaluated trials for biases, which included randomization process, deviations from intended interventions, missing outcome data, measurement of the outcome, selection of the reported outcome, and overall bias included in RCTs. The results extracted from each RCT were used until the consensus was reached between the two researchers. We resolved disagreements by discussing or negotiating with the third researcher (ZHL) until the consensus was reached.

### Data extraction

A self-designed standardized data extraction form was used to extract the data independently. The extracted information included the primary author, year of publication, sample size, surgical site, localization technique of nerve block, type and dose of LA, dose of dexmedetomidine, analgesic effects, postoperative pain scores, postoperative analgesic consumption, and dexmedetomidine-related side effects. Inconsistencies in the data extraction process were settled by rechecking the original data and consulting with the third researcher (ZHL).

Data were extracted from the tables as the first provenance for extraction. When the data were incomplete, we contacted the original author for more information. Also, we used an estimate of the standard deviation (SD), SD = Range/4 and SD = interquartile range (IQR)/1.35, to include trials in which the range and IQR were reported, as described by the Cochrane Handbook for Systematic Reviews (Cumpston et al., 2019). Data reported with 95% confidence intervals (CIs) were also used to estimate the range and converted to SD. If the mean was not provided, the median was used to estimate its value (Hozo et al., 2005). When the required data were present in the figures and the original data were not obtained from the authors, we extracted data from the published figures using ImageJ software (ImageJ software, National Institutes of Health, United States, http://imagej.nih.gov). Postoperative pain severity reported using the numerical rating scale scores was converted to visual analog scale (VAS) scores (Breivik et al., 2000).

### Outcomes assessed

We designated postoperative pain severity using VAS scores (0 = no pain; 10 = worst pain imaginable) at 12 and 24 h postoperatively as the primary outcome. Secondary outcomes were analgesic effects, and the indicators included duration of analgesia (min), cumulative analgesic drug consumption during the first 48 h postoperatively, frequency of adverse effects (Ebert et al., 2000) (bradycardia and hypotension), and postoperative nausea and vomiting (PONV).

### Predefined sources of heterogeneity

To explore the reasons for heterogeneity, the clinical characteristics of individual trials and known confounders that may have contributed to variation in the primary outcome (severity of postoperative pain) were pre-determined. These variables primarily included surgery type, nerve block localization technique, type and dose of LA, and dexmedetomidine dose. Based on the clinical hypothesis that different surgery types and LA types lead to different pain intensities and analgesic effects, we analyzed the results separately by the type of surgery and LA.

### Statistical analysis

One author (TR) performed the data entry, and another author (LYQ) checked its accuracy. The meta-analysis was performed using Review Manager (RevMan for Windows, Version 5.3) to combine the data, and for all time-to-event outcomes, including postoperative pain severity and duration of analgesia, we calculated the ratio of means, SD, and 95% CI for all continuous outcomes (Friedrich et al., 2012). For other results, dichotomous outcomes used ORs and 95% CIs, and continuous outcomes used weighted mean differences and 95% CIs. The differences were considered statistically significant when the *p*-value < 0.05.

I^2^ statistics were used to assess the heterogeneity of the combined results (Higgins and Thompson, 2002). Heterogeneity was significant (I^2^ > 50%), which indicated heterogeneity in the included studies. We selected random effect modeling to pool the results, and we performed subgroup analysis or meta-regression according to the characteristics of the included studies (surgical site) to find the sources of heterogeneity. When heterogeneity was not significant (I^2^ < 50%), we selected fixed effect modeling to pool the results. A funnel plot was created for multiple trial results by incorporating effect estimates from trials and their accuracy. The risk of publication bias was assessed by examining the asymmetry of funnel plots. If asymmetry was indicated by visual assessment, we investigated the cause of the funnel plot asymmetry using exploratory analysis (sine test for binary data and Egger’s test for continuous data). Furthermore, sensitivity analysis was performed by removing individual studies one at a time to examine the influence of the quality of the included studies on the results of the meta-analysis.

We assessed the strength of evidence collected from the included trials using the Grade of Recommendations, Assessment, Development, and Evaluation (GRADE) guidelines. The GRADE system exhaustively describes the factors that affect the quality of evidence and provides quantitative criteria for grading (Guyatt et al., 2011; Mendoza et al., 2017). Therefore, our study used the GRADE grading method (validity, consistency, precision, and applicability of results) to assess the quality of evidence. The GRADE tool classified the strength of combined evidence into four levels, namely, high quality, moderate quality, low quality, and very low quality.

## Results

**FIGURE 1:**
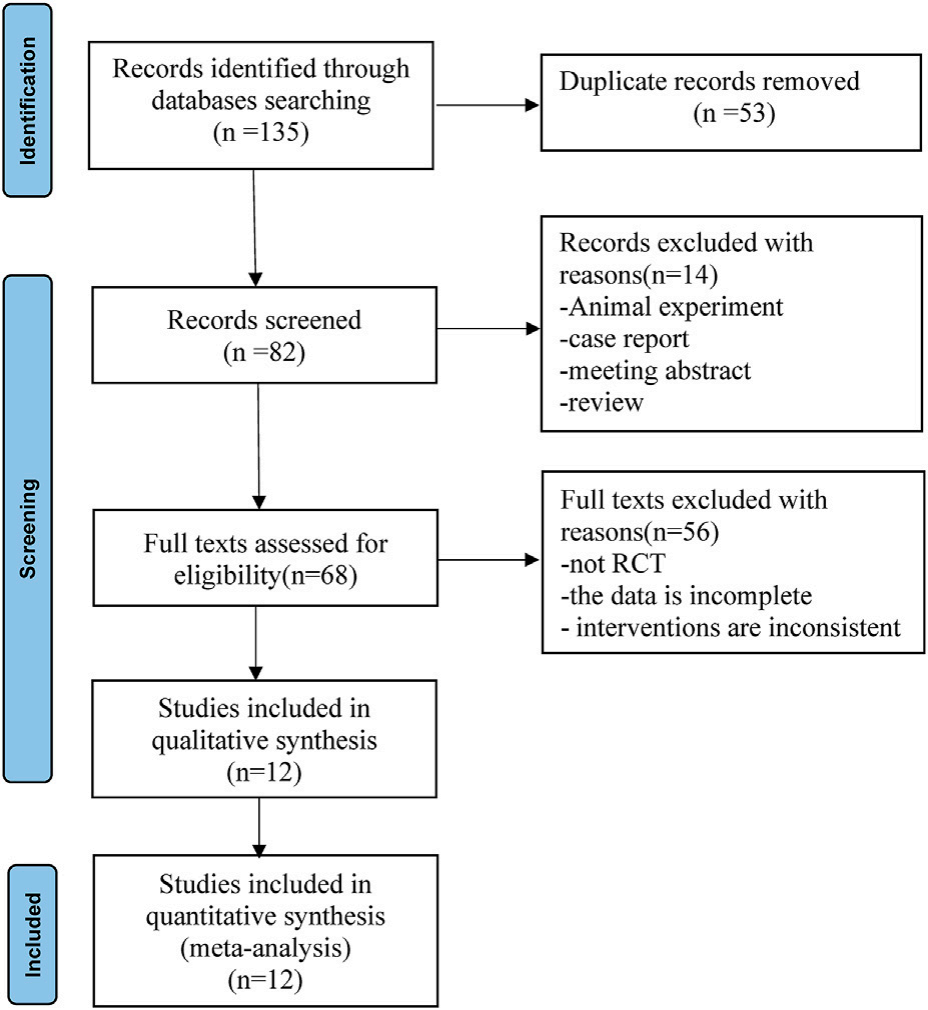
Flow diagram summarizing the retrieved, included, and excluded randomized trials.

According to the established retrieval strategy, 135 relevant published records were retrieved from the database. Eighty-two of these records were obtained after deleting duplicates. Preliminary screening of titles and abstracts excluded 14 records that did not conform to the inclusion criteria. After reviewing the full text, 56 records were excluded because the study was not an RCT, the data were incomplete, or the interventions were inconsistent. A total of 12 full-text randomized trials (Mohamed et al., 2014; Chen et al., 2015; Tian et al., 2015; Mohta et al., 2016; Dutta et al., 2017; Jin et al., 2017; Ding et al., 2018; Xu et al., 2018; Abd-Elshafy et al., 2019; Alimian et al., 2021; Sen et al., 2021; Zha et al., 2021) ultimately met the inclusion criteria for meta-analysis. The flowchart for the retrieval and filtering of records is shown in Figure 1 and summarizes the reasons for the exclusion of records. No other eligible studies were found after a manual supplemental search.

### Trial characteristics

A total of 701 subjects were included in this meta-analysis. There were 328 subjects in the control group and 326 subjects in the dexmedetomidine group. The details of the 12 RCTs, surgical sites, interventions, sample sizes, and assessments of the primary outcomes are summarized in Table 1. The nerve block localization methods were anatomical localization in one trial (Mohamed et al., 2014), ultrasound localization in 10 trials (Chen et al., 2015; Tian et al., 2015; Dutta et al., 2017; Jin et al., 2017; Ding et al., 2018; Xu et al., 2018; Abd-Elshafy et al., 2019; Alimian et al., 2021; Sen et al., 2021; Zha et al., 2021), and not defined in one trial (Mohta et al., 2016). All trials used long-acting LAs (ropivacaine, bupivacaine, or levobupivacaine) alone. Only one trial used a fixed dose of dexmedetomidine (100 µg) (Alimian et al., 2021), and the rest of the trials used dexmedetomidine based on weight. Analgesic outcomes were reported in all trials.

**TABLE 1:** Trial characteristics and outcomes examined.

Author	Sample size	Surgery	Group	LA concentration—total volume	Dex dose	Block localization	Primary outcome
Abd-Elshafy et al. (2019)	60	VATS surgery	1. Bupivacaine + NS(30)	0.5%—0.3 ml/kg	1 µg/kg	Ultrasound	Pain scores at 2 h, 4 h, 8 h, and 24 h
			2. Bupivacaine + Dex (30)				
Alimian et al. (2021)	42	Laparotomy	1. Bupivacaine + NS(21)	0.25%—20 ml	100 µg	Ultrasound	Pain scores at 2 h, 6 h, 12 h, 24 h, and 48 h
			2. Bupivacaine + Dex (21)				
Ding et al. (2018)	102	Thoracoscopic lobectomy	1. Ropivacaine + NS(36)	0.5%—15 ml	1 µg/kg	Ultrasound	Pain scores at 2 h, 6 h, 12 h, 24 h, 36 h, and 48 h
			2. Ropivacaine + Dex (34)				
			3. TEA (32)*				
Dutta et al. (2017)	30	Thoracic surgery	1. Ropivacaine + NS(15)	0.75%—15 ml	1 µg/kg	Ultrasound	Pain scores at 2 h, 4 h, 8 h, 12 h, and 24 h
			2. Ropivacaine + Dex (15)				
Tian et al. (2015)	60	Thoracic surgery	1. Ropivacaine + NS(30)	0.375%—20 ml	1 µg/kg	Ultrasound	Sensory block onset time and duration
			2. Ropivacaine + Dex (30)				
Jin et al. (2017)	72	Breast cancer surgery	1. Bupivacaine + NS(36)	0.25%—20 ml	1 µg/kg	Ultrasound	Pain scores at 2 h, 6 h, 12 h, 24 h, 36 h, and 48 h
			2. Bupivacaine + Dex (36)				
Mohamed et al. (2014)	60	Modified radical mastectomy	1. Bupivacaine + NS(30)	0.25%—20 ml	1 µg/kg	Landmark	Pain scores at 2 h, 6 h, 12 h, 24 h, 36 h, and 48 h
			2. Bupivacaine + Dex (30)				
Mohta et al. (2016)	45	Modified radical mastectomy	1. Bupivacaine + NS(15)	0.5%—0.3 ml/kg	1 µg/kg	Not defined	Pain scores at 2 h, 4 h, 8 h, 12 h, and 24 h
			2. Bupivacaine + Dex (15)				
			3. Normal saline (15)*				
Sen et al. (2021)	70	Laparoscopic cholecystectomy	1. Bupivacaine + NS(35)	0.25%—15 ml	1 µg/kg	Ultrasound	Pain score at an unspecified time point
			2. Bupivacaine + Dex (35)				
Xu et al. (2018)	60	VATS surgery	1. Ropivacaine + NS(30)	0.375%—20 ml	1 µg/kg	Ultrasound	Pain scores at 2 h, 6 h, 12 h, 24 h, 36 h, and 48 h
			2. Ropivacaine + Dex (30)				
Zha et al. (2021)	60	VATS surgery	1. Ropivacaine + NS(20)	0.5%—10 ml	1 µg/kg	Ultrasound	Pain scores at 4 h, 8 h, 12 h, and 24 h
			2. Ropivacaine + Dex (20)				
			3. Routine general anaesthesia (20)*				
Chen et al. (2015)	40	Thoracic surgery	1. Ropivacaine + NS(20)	0.5%—15 ml	0.75 µg/kg	Ultrasound	Sensory block onset time and duration
			2. Ropivacaine + Dex (20)				

Abbreviations: Dex, dexmedetomidine; LA, local anesthetic; CI, confidence interval; N, number; VATS, video-assisted thoracoscopic surgery.

### Risk of bias assessment

**FIGURE 2:**
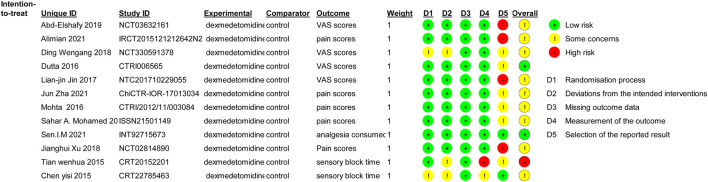
Risk of bias summary. Review authors’ judgments about each risk of bias item for each included study. Green circle, low risk of bias; red circle, high risk of bias; yellow circle, unclear risk of bias.

The reviewers’ consensus assessment of the included trials is shown in the risk bias chart in Figure 2. Random assignment procedures were clearly reported in some trials, but some of the trials lacked sufficient details to fully assess the risk of bias. When the details of the randomization process and allocation sequence concealment were not available, we were conservative in the degree of risk of bias assessment and preferred to classify the study as “some concerns.” Studies that used generalized descriptions, such as “similar side effects between study groups,” or reported hemodynamic outcome data in a graphical format, which cannot determine the actual risk of the side effects, were rated as high risk for “selection of the reported result.” Our evaluation criteria were relatively conservative, and the primary outcomes (postoperative pain severity) were unlikely to be affected by the aforementioned biases. Therefore, we considered the methodological quality of the 12 included trials to be acceptable and assessed the overall risk of bias of the included trials as moderate. The risk of a random process, deviations from the intended interventions, missing outcome data, and measurement of the outcome were low risks in most trials. Due to the aforementioned conservative approach used to assess hemodynamic side effects associated with dexmedetomidine, the selection of the reported result was high in some trials.

### Postoperative pain scores

**FIGURE 3:**
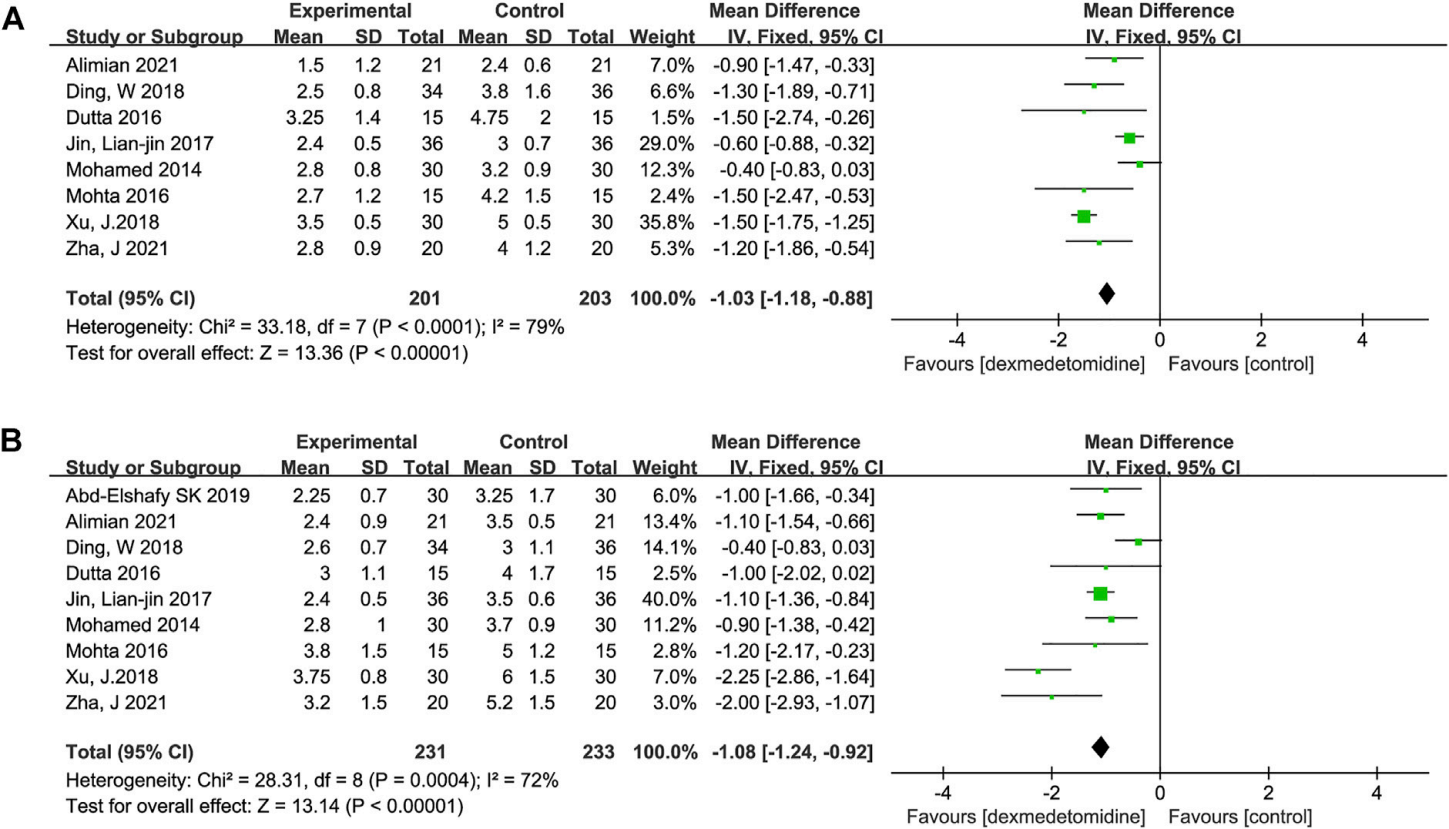
Forest plots of postoperative pain scores. SD, standard deviation; CI, confidence interval. **(A)** Forest plots of postoperative pain scores at 12 h after surgery. **(B)** Forest plots of postoperative pain scores at 24 h after surgery.

Data describing the primary outcome, postoperative pain severity (postoperative pain scores), were obtained at 12 h in eight trials (Mohamed et al., 2014; Mohta et al., 2016; Dutta et al., 2017; Jin et al., 2017; Ding et al., 2018; Xu et al., 2018; Alimian et al., 2021; Zha et al., 2021) and at 24 h in nine trials (Mohamed et al., 2014; Mohta et al., 2016; Dutta et al., 2017; Jin et al., 2017; Ding et al., 2018; Xu et al., 2018; Abd-Elshafy et al., 2019; Alimian et al., 2021; Zha et al., 2021). We used the random effect model and took MD as the outcome index. The meta-analysis showed that the application of dexmedetomidine as a PVB adjunct reduced the postoperative pain severity of patients 12 and 24 h after surgery compared to the control group. Expressed as MD (95% CI), the results were −1.03 (−1.18, −0.88) (*p* < 0.00001, I^2^ = 79%) for 12 h (Figure 3A) and −1.08 (−1.24, −0.92) (*p* < 0.00001, I^2^ = 72%) for 24 h (Figure 3B). The primary outcomes were significantly heterogeneous. Therefore, subgroup analysis was performed according to the surgery type and LA type.

For subgroup analysis based on the surgery type, there was only one study on laparotomy surgery (Alimian et al., 2021), and statistical significance could not be analyzed. The other types of surgery were VATS, thoracic surgery, and breast surgery; the yielded omnibus *p*-values were 0.40, 0.77, and 0.12 for 12 h pain scores, which suggested that there were no significant differences in the subgroup analysis of pain scores at 12 h postoperatively. However, in the same subgroup analysis of 24 h pain scores, we found that the yielded omnibus *p*-values were 0.00001, 0.84, and 0.22, which indicated that the VATS may be the source of heterogeneity of pain scores 24 h after surgery. The combined results are presented in Table 2.

**TABLE 2:** Summary of subgroup analysis of dexmedetomidine on postoperative pain scores at 12 and 24 h.

Primary outcome	Subgroup	Number of studies included	References of studies	Dex N	Control N	Weighed mean (95% Cl)	*p*-value for statistical significance	*p*-value for heterogeneity	I^2^ test for heterogeneity (%)
Pain scores at 12 h	LA type								
	Bupivacaine	4	22, 24, 25, and 29	102	102	−0.68 (−1.00, −0.37)	<0.0001	0.17	41
	Ropivacaine	4	23, 27, 30, and 31	99	101	−1.44 (−1.66, −1.22)	<0.00001	0.81	0
	Surgery type								
	VATS	2	27 and 30	50	50	−1.46 (−1.70, −1.23)	<0.00001	0.4	0
	Thoracic surgery	2	23 and 31	49	51	−1.34 (−1.87, −0.81)	<0.00001	0.77	0
	Breast surgery	3	24, 25, and 29	81	81	−0.64 −1.04, −0.25)	0.001	0.13	51
Pain scores at 24 h	LA type								
	Bupivacaine	5	22, 24, 25, 28, and 29	132	132	−0.57 (−0.93, −0.21)	0.002	0.03	63
	Ropivacaine	4	23, 27, 30, and 31	99	101	−1.30 (−2.47, −0.13)	0.03	<0.00001	95
	Surgery type								
	VATS surgery	3	27, 28, and 30	80	80	−2.13 (−4.03, −0.23)	0.03	<0.00001	95
	Thoracic surgery	2	23 and 31	49	51	−0.40 (−0.80, −0.000	0.05	0.84	0
	Breast surgery	3	24, 25, and 29	81	81	−0.44 (−0.84, −0.05)	0.03	0.22	34

Abbreviations: Dex, dexmedetomidine; TEA, thoracic epidural anaesthesia; NS, normal saline; VATS, video-assisted thoracoscopic surgery; kg, kilogram; ml, milliliter; µg, microgram; h, hour; (*) excluded from the analysis.

For subgroup analysis based on the LA type, we included bupivacaine and ropivacaine. The combined results were -0.68 (−1.00, −0.37) (I^2^ = 41%, *p* = 0.17) and −1.44 (−1.66, −1.22) (I^2^ = 0%, *p* = 0.81) for 12 h pain scores, which suggested that there were no significant differences in the subgroup analysis of pain scores at 12 h postoperatively. However, the results of the subgroup analysis for 24 h pain scores were −0.57 (−0.93, −0.21) (I^2^ = 63%, *p* = 0.03) and −1.30 (−2.47, −0.13) (I^2^ = 95%, *p* < 0.00001), as shown in Table 2. It indicated that the LA type may be the source of heterogeneity of pain scores 24 h after surgery.

### Analgesic outcomes

The effect of dexmedetomidine as an adjunct to PVB on the postoperative analgesia duration was assessed in 10 trials (Mohamed et al., 2014; Chen et al., 2015; Tian et al., 2015; Mohta et al., 2016; Jin et al., 2017; Ding et al., 2018; Abd-Elshafy et al., 2019; Alimian et al., 2021; Sen et al., 2021; Zha et al., 2021). The duration of postoperative analgesia was defined as the time to first analgesia request. The meta-analysis showed that the use of dexmedetomidine as a PVB adjunct prolonged the duration of analgesia by at least 173.27 min (115.61, 230.93) (*p* < 0.00001, I^2^ = 81%), as shown in Table 3.

**TABLE 3:** Summary of secondary outcomes.

Analgesic outcome	Number of studies included	References of studies included	Dex *N*	Control *N*	Weighed mean (95% CI)	*p*-value for statistical significance	*p*-value for heterogeneity	I^2^ test for heterogeneity
Duration of analgesia (min)	9	22-29, 32, and 33	271	273	173.27 (115.61, 230.93)	<0.00001	<0.00001	81%
Analgesic consumption	5	22, 24, 25, 27, and 29	122	122	−18.01 (−22.10, −13.92)	<0.00001	0.29	19%
Dex-related adverse effect	Number of studies included	References of studies included	Dex n/N (%)	Control n/N (%)	Odds ratio (95% Cl)	*p*-value for statistical significance	*p*-value for heterogeneity	I^2^ test for heterogeneity
Nausea and vomiting	8	23–27, 30, 32, and 33	34/235 (14.5%)	27/237 (11.4%)	1.30 (0.76, 2.20)	0.34	0.04	53%
Hypotension	3	23, 30, and 31	7/79 (8.9%)	6/81 (7.4%)	1.22 (0.40, 3.72)	0.72	0.67	0%
Bradycardia	2	23 and 30	2/45 (4.4%)	0/45 (0%)	3.15 (0.31, 31.61)	0.33	0.99	0%

Abbreviations: Dex, dexmedetomidine; N, number; CI, confidence interval.

Five trials (Mohamed et al., 2014; Mohta et al., 2016; Jin et al., 2017; Alimian et al., 2021; Zha et al., 2021) reported cumulative analgesic consumption after surgery; cumulative analgesic consumption 48 h after surgery was expressed as oral morphine equivalent. The analysis results are shown in Table 3. Dexmedetomidine combined with LA reduced oral morphine equivalent consumption by an MD (95% CI) of −18.01 mg (−22.10, 13.92) (*p* < 0.00001, I^2^ = 19%).

### Adverse effects

The most frequently reported adverse effects in all trials were nausea and vomiting, hypotension, and bradycardia. With 0*R* as the outcome index, the obtained analysis result was 1.30 (0.76, 2.20) (*p* = 0.34, I^2^ = 53%), which indicated no significant differences in nausea and vomiting between the two groups.

Although dexmedetomidine may increase the incidence of hypotension and bradycardia, we found that the obtained analysis results of hypotension and bradycardia were 1.22 (0.40, 3.72) (*p* = 0.72, I^2^ = 0%) and 3.15 (0.31, 31.61), (*p* = 0.33, I^2^ = 0%), respectively. This finding indicated no statistically significant differences in hemodynamic complications between the two groups (Table 3).

### The risk of publication bias and sensitivity analysis

**FIGURE 4:**
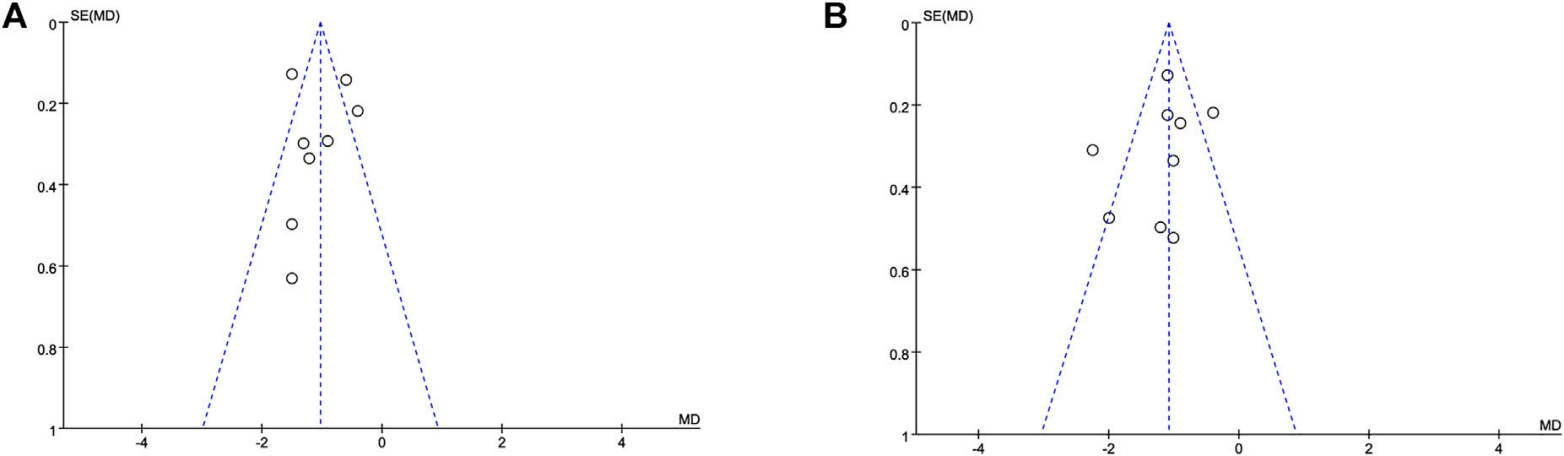
Funnel plot based on the pain scores. Abbreviations: MD, mean difference. **(A)** Funnel plot based on the pain scores at 12 h. **(B)** Funnel plot based on the pain scores at 24 h.

A funnel plot based on the pain scores at 12 h (Figure 4A) and 24 h (Figure 4B) between the dexmedetomidine group and the control group showed that the distribution of each study on both sides of the funnel plot was symmetrical, which suggested no publication bias. Furthermore, sensitivity analysis was performed by removing individual studies one at a time, and the results showed that the direction of effect size combination results did not change, which suggested that the meta-analysis results were stable.

### Grade of recommendations, assessment, development, and evaluation rating of outcome indicators

**FIGURE 5:**
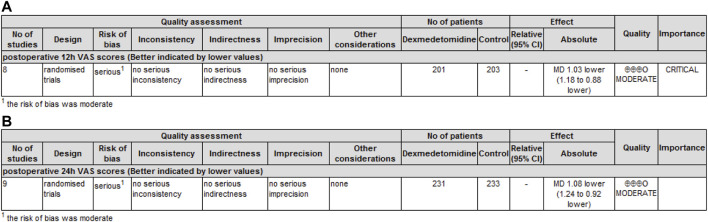
GRADE rating for evidence of pain scores. Abbreviations: VAS, visual analog scale; MD, mean difference. **(A)** GRADE rating for evidence of pain scores at 12 h. **(B)** GRADE rating for evidence of pain scores at 24 h.

GRADE rating was performed for primary and secondary outcomes. We found that the level of evidence for postoperative pain scores at 12 h (Figure 5A) and 24 h (Figure 5B) was rated as moderate. The risk of bias reduced the overall quality evaluation.

**FIGURE 6:**
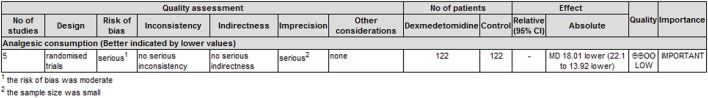
GRADE rating for evidence of the duration of analgesia and cumulative analgesic consumption. Abbreviations: MD, mean difference.

**FIGURE 7:**
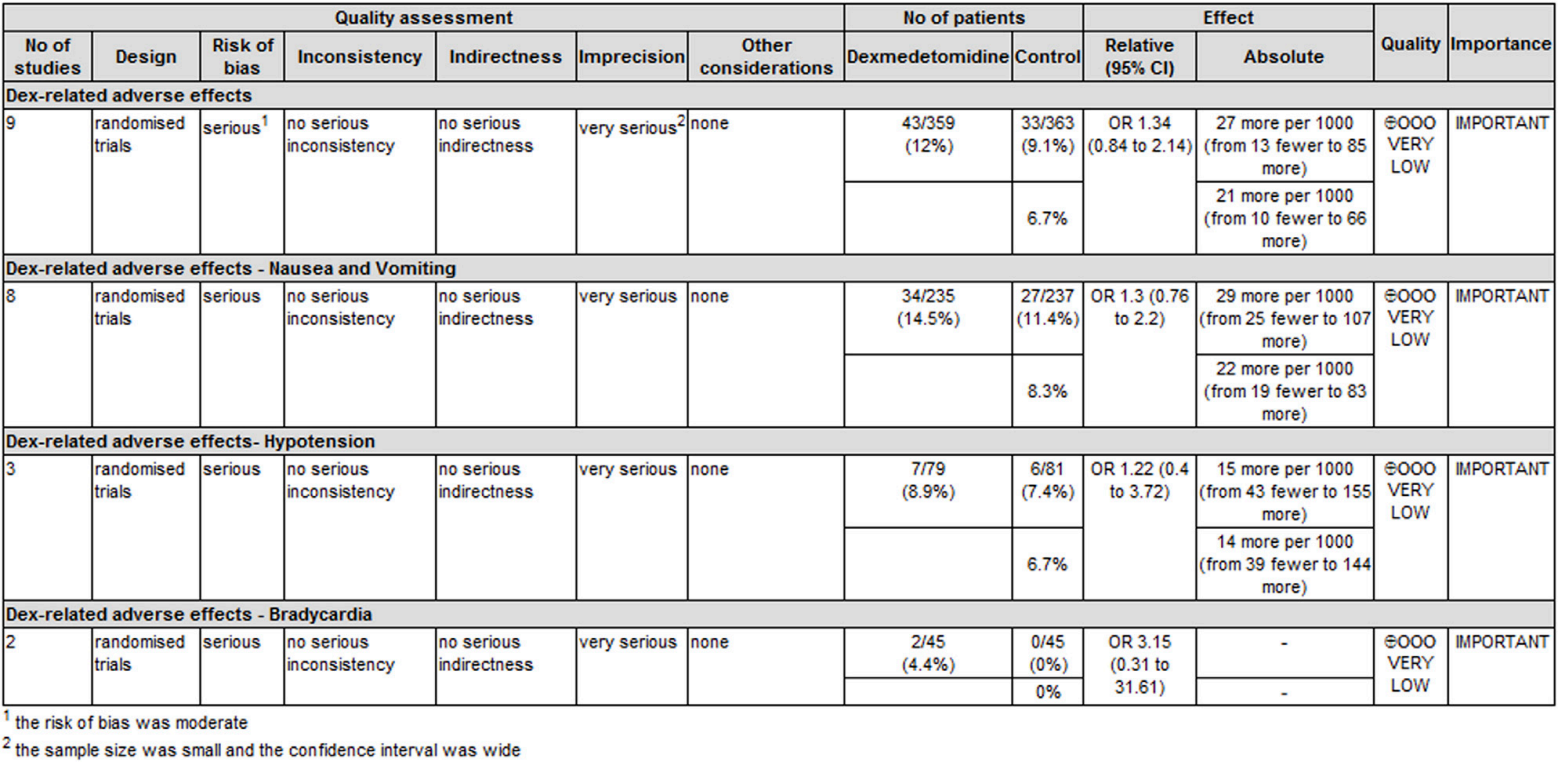
GRADE rating for evidence of adverse effects. Abbreviations: OR, odds ratio.

The level of evidence for the duration of analgesia and postoperative analgesic consumption was rated as low because the risk of bias and imprecision downgraded the overall quality assessment (Figure 6). The level of evidence for dexmedetomidine-related adverse effects was rated as very low primarily because the risk of bias and imprecision downgraded the quality assessment (Figure 7).

## Discussion

The present study provides evidence from clinical trials supporting the efficacy of dexmedetomidine as an adjunct to PVB. The results of this meta-analysis indicated that using dexmedetomidine with LA for PVB significantly improved postoperative pain scores, prolonged the duration of analgesia, and reduced postoperative analgesic consumption compared to LA alone. These results are similar to a previous study evaluating dexmedetomidine as an adjunct for brachial plexus block (Vorobeichik et al., 2017).

Our results showed that dexmedetomidine reduced pain scores by 1.03–1.08 at 12 and 24 h after surgery and prolonged the duration of analgesia by at least 173.27 min. The results were similar to the meta-analysis published in 2018 (Wang et al., 2018), which showed that dexmedetomidine reduced pain scores by 0.86 (*p* = 0.01, I^2^ = 96%) and 0.93 (*p* = 0.008, I^2^ = 97%) at 12 and 24 h postoperatively, respectively. However, the results of this study were characterized by high heterogeneity. Although these researchers performed further subgroup and sensitivity analyses to identify the origin of heterogeneity, they unfortunately failed to identify the source. In comparison, the results of our study also had significant heterogeneity in the primary outcome, but the subgroup analysis in our study showed that LA type and VATS may be the source of heterogeneity in pain scores 24 h after surgery. The result implied that the effect of dexmedetomidine was different in combination of different types of LA, especially when dexmedetomidine was combined with ropivacaine, the pain scores seemed to decrease even more after surgery. Although we are not clear about the relevant mechanism yet, it can provide clinicians with a more convenient and quick access tool when making medical decisions. Second, dexmedetomidine combined with LA had a better effect on reducing postoperative pain scores after VATS primarily because the VATS had smaller incision, less trauma, and nerve damage. The levels of evidence correlated with postoperative pain scores were moderate. These results provide a solid foundation for a more detailed evaluation of dexmedetomidine combined with LA in PVB in the future. However, the present results were also highly heterogeneous. Although the type of surgery and LA accounted for some of the heterogeneity, our findings must be treated with caution.

Transient nausea and vomiting, hypotension, and bradycardia were associated with perineural dexmedetomidine, but there were no statistically significant differences in the incidence of adverse effects between the two groups in our study. These results are different from previous studies (Chen et al., 2015; Wang et al., 2018; Xu et al., 2018), primarily due to the small sample size of individual studies, which magnified the effect of adverse reactions. The use of dexmedetomidine should be weighed against enhanced pain relief and the potential risk of side effects. Some adverse reactions may interfere with patient’s enhanced recovery after surgery or other pathways of expediting discharge from the hospital. Potential hemodynamically related side effects may limit the use of dexmedetomidine because bradycardia and hypotension may be easily identified by existing monitoring systems. These adverse reactions may limit the use of dexmedetomidine in patients with underlying cardiac disease.

The results of this meta-analysis indicated that dexmedetomidine had important clinical value in peripheral nerve block. Keplinger et al. (2015) studied the pharmacokinetics and pharmacodynamics of dexmedetomidine combined with ropivacaine in peripheral nerve block, and the results showed that 100 μg dexmedetomidine combined with ropivacaine was the optimal dose for peripheral nerve block. A case report of a lower limb amputation with dexmedetomidine combined with ropivacaine for sciatic nerve block in a patient with cardiovascular disease found that the block time was extended by 26 h (Wang et al., 2015). Helal et al. (2016) investigated the effect of dexmedetomidine combined with bupivacaine on sciatic and femoral nerve block and found that patients who received dexmedetomidine in combination with bupivacaine anaesthesia had a 20% shorter time of anaesthesia recovery and sensory and motor recovery those who received bupivacaine alone. The duration of sensory and motor nerve block increased by 45% and 40%, respectively, and the duration of analgesia increased by 75%. Sciatic nerve block was performed in animals with high doses of dexmedetomidine (20–40 mg/kg) combined with bupivacaine (Brummett et al., 2008) and ropivacaine (Brummett et al., 2009), and no neurotoxicity or axonal or myelin injury was found 24 h and 14 days after injection. The results of *in vitro* and animal studies suggested that the peripheral use of dexmedetomidine had a neuroprotective effect on LA-induced inflammatory responses (Brummett et al., 2008; Tüfek et al., 2013; Huang et al., 2014). Most of the studies are animal experiments and *in vitro* neural experiments, and there is no strong evidence of toxicity to human peripheral nerve fibers in clinical application.

The improvement in clinical efficacy in the dexmedetomidine group may be caused by the peripheral mechanism of action of dexmedetomidine or the central effects of absorption and systemic redistribution. Dexmedetomidine is an α2-adrenergic agonist that acts on α2 receptors in the locus coeruleus to produce sedative and hypnotic effects and produces analgesic effects by acting on α2 receptors in the locus coeruleus and spinal cord (Guo et al., 1996). The analgesic time of dexmedetomidine combined with ropivacaine was prolonged by approximately 75% compared to ropivacaine alone. The analgesic effect of dexmedetomidine was not reversed after the administration of an α2 receptor antagonist. The results indicated that the analgesic effect of dexmedetomidine occurred *via* a peripheral mechanism rather than a central effect of systemic redistribution (Brummett et al., 2011). Fritsch et al. (2014) measured plasma levels of 150 µg dexmedetomidine combined with ropivacaine for intermuscular brachial plexus block and confirmed that the prolonging effect of dexmedetomidine on the duration of analgesia was not systemic. The peripheral analgesic mechanism of dexmedetomidine is likely related to the reduction of norepinephrine release and inhibition of nerve fiber action potential *via α*2 receptors (Mohta et al., 2016).

There are several limitations to our review. First, the included studies were highly heterogeneous, and the source of heterogeneity could not be completely determined by subgroup analysis because of the limited data provided by the original trials. Second, the definitions and assessments of some outcomes were inconsistent between trials, which may be the reason for the heterogeneity. Third, the small sample size of the included trials increased the opportunity for type I errors and publication bias. Fourth, methodological deficiencies in the included trials and inconsistencies in the definition and assessment of outcomes were the main reasons for downgrading the strength of evidence for some outcomes. Although there were inconsistencies, the methods used to evaluate the efficacy of dexmedetomidine (postoperative pain scores and duration of analgesia) had good internal and external validity. Last, adverse reactions should also be considered in determining whether dexmedetomidine should be used for perineural or systemic treatment, and the long-term safety and mechanism of dexmedetomidine perineural administration must be further studied.

In contrast, there were several positives in our research. Our literature search was relatively comprehensive and included the most relevant databases. Our included trials were limited to RCTs. Despite our attempts to explore statistical heterogeneity, the main results remain robust. All of these advantages support the validity of our results.

## Conclusion

In summary, our study concluded that appropriate unilateral surgical procedures using dexmedetomidine combined with LA in PVB significantly improved postoperative analgesia. However, we cannot ignore the large heterogeneity between the studies in this meta-analysis. More large-scale prospective studies are needed to further clarify this conclusion.

## Data Availability

The original contributions presented in the study are included in the article/Supplementary Material; further inquiries can be directed to the corresponding authors.
